# Influence of type I collagen polymorphisms and risk of anterior cruciate ligament rupture in athletes: a case-control study

**DOI:** 10.1186/s12891-022-05105-2

**Published:** 2022-02-16

**Authors:** Jamila Alessandra Perini, Lucas Rafael Lopes, João Antonio Matheus Guimarães, Rodrigo Araújo Goes, Luiz Fernando Alves Pereira, Camili Gomes Pereira, Marcelo Mandarino, Alfredo Marques Villardi, Eduardo Branco de Sousa, Victor Rodrigues Amaral Cossich

**Affiliations:** 1 Divisão de Ensino e Pesquisa, Instituto Nacional de Traumatologia e Ortopedia (INTO), Avenida Brasil, 500, Rio de Janeiro, 20940-070 Brazil; 2 Laboratório de Pesquisa de Ciências Farmacêuticas, Centro Universitário Estadual da Zona Oeste (UEZO), Rio de Janeiro, Brazil; 3 Programa de Pós-graduação em Saúde Pública e Meio Ambiente, Escola Nacional de Saúde Pública, Fundação Oswaldo Cruz (Fiocruz), Rio de Janeiro, Brazil; 4 Centro de Trauma do Esporte, Instituto Nacional de Traumatologia e Ortopedia (INTO), Rio de Janeiro, Brazil; 5 Centro de Cirurgia do Joelho, Instituto Nacional de Traumatologia e Ortopedia (INTO), Rio de Janeiro, Brazil

**Keywords:** Collagen, ACL rupture, Polymorphisms, Athletes

## Abstract

**Background:**

Anterior cruciate ligament (ACL) rupture is a common and severe knee injury in sports and occurs mostly due to noncontact injuries. There is an increasing amount of evidence associating ACL rupture to single nucleotide polymorphisms (SNPs), and SNPs in the collagen type I genes can change its expression and tissue mechanical features. This study aimed to investigate the association between SNPs in *COL1A1* and *COL1A2* with sports-related ACL tears.

**Methods:**

A total of 338 athletes from multiple sports modalities were analyzed: 146 were diagnosed with ACL rupture or underwent an ACL reconstruction surgery and 192 have no musculoskeletal injuries. SNPs were genotyped using validated TaqMan assays. The association of the polymorphisms with ACL rupture was evaluated by a multivariable logistic regression model, using odds ratios (OR) and 95% confidence intervals (CI).

**Results:**

The age, sport modality, and training location were associated with an increased risk of a non-contact ACL tear. *COL1A2* SNPs (rs42524 *CC* and rs2621215 *GG*) were associated with an increased risk of non-contact ACL injury (6 and 4-fold, respectively). However, no significant differences were detected in the distribution of *COL1A1 rs*1107946 and *COL1A2* rs412777 SNPs between cases and controls. There was a protective association with ACL rupture (OR = 0.25; 95% CI = 0.07–0.96) between *COL1A1* rs1107946 (*GT* or *TT*) and the wildtype genotypes of the three *COL1A2* (rs412777*,* rs42524*,* rs2621215). *COL1A2* rs42524 and rs2621215 SNPs were associated with non-contact ACL risk.

**Conclusion:**

The combined analysis of *COL1A1*-*COL1A2* genotypes suggests a gene-gene interaction in ACL rupture susceptibility.

## Background

The ligaments are mechanical restraints that help on the joint normal kinematics and stability [1, 2]. In this context, the anterior cruciate ligament (ACL) is the main mechanical restrictor of excessive tibia anterior translation [3, 4] and prevents knee excessive external rotation and varus-valgus stresses, particularly under bodyweight bearing [5, 6]. Hence, the ACL is crucial for the knee proper function, and its rupture leads to acute joint functional instability – anteroposterior and rotational laxity – functional symptoms, and other intra-articular structures damage [7]. Consequently, resulting in joint effusion, muscle weakness, altered movement pattern, and reduced functional performance [8].

ACL rupture is a very common and severe knee injury in sports and occur mostly due to noncontact injuries [9], being the most common mechanisms of a deceleration event together with a sudden change in direction with a planted and/or a non-compensated dynamic valgus during landing [10, 11]. It makes the ACL tears of huge concern in sports involving jumping, pivoting, and cutting maneuvers [12, 13].

The treatment after an ACL rupture is mainly surgical, and the rehabilitation process is a time-consuming effort, which can endure up to 9–12 months for complete healing and return-to-sport [14, 15]. Nevertheless, even when treated properly there are odds for a second rupture, and development of concomitant and chronic degenerative articular injuries (e.g., meniscus tears, osteoarthritis) [16]. If it was not enough, surprisingly only 65% of soccer players return to participate at the same pre-injury level 3 years after the ACL tear [17]. Altogether those pieces of evidence highlight that an ACL tear is a social, economic, and professional burden for sports teams and the affected athlete [18]. Thus, a better understanding of the extrinsic – type and level of activity, playing surface, and equipment used – and intrinsic – sex, hormonal, anatomical, neuromuscular, and genetic contribution – risks factors is mandatory for designing target injury preventive interventions [9, 19, 20].

There is an increasing amount of evidence that genetic sequence variants play an important role in ACL rupture occurrence [21, 22] and single nucleotide polymorphisms (SNPs) in the collagen genes have already been associated with a genetic susceptibility for ACL rupture [20, 23–28]. What makes sense, since the ACL is a multi-fascicular structure – a built of dense and regular connective tissue mainly of collagen (~ 75% of its dry weight) – predominantly of type I (up to 85% of ACL’s collagen) [29, 30], which is believed it to be responsible for the ligament tensile strength [6, 29].

Type I collagen consists of two α1 and one α2 chains, encoded by the genes *COL1A1* (chromosome 17q21) and *COL1A2* (7q21.3), respectively. SNPs on those genes can change type I collagen expression and tissue mechanical features [31] and, consequently, affect human tissue development and repair [32, 33]. For example, the *COL1A1* rs1107946 SNP located in the promoter region (*− 1997G > T*) showed higher transcriptional activity, influencing gene expression regulation in the osteoblasts [34]. Also, SNPs in the *COL1A2* gene at the coding or non-coding regions (rs412777, rs42524, and rs2621215) and their possible haplotypes can influence the type I collagen function [31] and, consequently, change the protein structure [32].

To the best of our knowledge, no studies evaluated the genetic combination of the *COL1A1* and *COL1A2* polymorphisms with susceptibility to ACL rupture. Thus, this study aimed to investigate the association between SNPs in both genes with sports-related ACL tears in athletes. The epidemiological, clinical, and athletic profiles were also explored to better understand the extrinsic and intrinsic ACL rupture risk factors in Brazilian athletes.

## Methods

### Participants

A total of 338 athletes between 18 and 45 years old from multiple sports modalities were prospectively selected for the present study. The subjects diagnosed with ACL rupture – only cases confirmed by clinical and magnetic resonance image – or who underwent an ACL reconstruction surgery previously were selected to compose the case group (*N* = 146). While the control group (*N* = 192) was composed of subjects without evidence of musculoskeletal injuries. Only subjects with complete orthopedic data and biological material collected were included in the present study. All subjects provided informed consent and the research was approved by the local Ethics Committee (#2.455.630/2017). The study was conducted in accordance with the Helsinki Declaration.

### Experimental approach

This was a case-control study. The volunteers were recruited between March 2018 and July 2019 at different sports training centers, sports competitions events, and the sports trauma outpatients service at our institution. First, the athletes answered the previously validated questionnaire to assess the general, training, and injuries information [35]. Follow a non-invasive sample from the oral mucosa was collected for genotyping analyses of collagen type I genes.

### Musculoskeletal injuries questionnaire

Besides the data regarding musculoskeletal injuries’ history, also was acquired clinical ACL tears-specific information as if the injury occurred during an official sports event, by contact or non-contact, report of post-training knee pain, ACL reconstruction surgery, injury recurrence, and time away from training due to the lesion, competitive practice (time and level), years of training, and weekly training hours. The data was collected with a printed version of the questionnaire “Musculoskeletal injuries report for Brazilian athletes” [35], immediately checked by the researcher who applied it with the athlete to clarify doubts and increase data reliance, and then the answers were transferred to a computational database and double-checked by different trained researchers.

### Polymorphism genotyping

Athletes’ genomic DNA was collected using a swab from the oral mucosa and isolated using an extraction kit (Qiagen, Hilden, Germany). Genotyping analyses of *COL1A1* (rs1107946) and *COL1A2* (rs412777, rs42524, rs2621215) SNPs were performed using TaqMan allelic discrimination assays (Table 1). For all polymorphisms, real-time polymerase chain reaction (PCR) was performed on a QuantStudio™ 3 Real-Time PCR System (Thermo Fisher Scientific, Waltham, MA USA). PCR amplification was performed in 8 μL reactions with 1 μL of template DNA (3–23 ng/μl), 1× TaqMan Universal Master Mix, 1× each primer and probe assay. Thermal cycling was initiated with a first denaturation step of 10 min at 95 °C, followed by 40 cycles of denaturation at 92 °C for 15 s and annealing at 60 °C for 1 min. To assure genotyping quality, in each reaction two standardized positive controls of each polymorphism genotype were used, as previously described [36].

**Table 1:** Characterization of *COL1A1* and *COL1A2* polymorphisms and probes for genotyping by TaqMan real-time PCR

Gene	Identified SNP	Position	Location	TaqMan Assays	Probe [SNP]
*COL1A1*	rs1107946	*−1997 G > T*	PR	C___7477171_10	CCTACTGTGGGTCAGTTCCAAGAGA **[A/C]** CCCCTCCCTAATAGGCGACAGGGGT
*COL1A2*	rs412777	*19,276,090 A > C*	Exon 25	C___2545507_10	ACGGCAGGCCTGGCCCAATTGGCCC **[A/C]** GCTGGAGCAAGAGGAGAGCCTGGCA
*COL1A2*	rs42524	*19,277,392 G > C*	Exon 28	C___2258177_20	AGGTGGAAAAGGTGAACAGGGTCCC **[C/G]** CTGGTCCTCCAGGCTTCCAGGTAAG
*COL1A2*	rs2621215	*19,290,197 T > G*	Intron 46	C__16038824_10	GTGAGGATTAAGGGAGATAGAAATA**[G/T]** ACATACAATAAAATCTCCTGGTAAC

PR is Promoter region

### Statistical analysis

The data distribution was verified by the Shapiro-Wilk test. The case athletes were subdivided according to contact or non-contact ACL rupture. Continuous variables were reported as mean ± SD and differences between all ACL rupture cases and controls groups were checked using the student’s t-test. However, according to their distribution and clinical significance, for the analysis, continuous variables (age, years of training, and weekly training hours) were divided into quartiles. The nominal data were shown in proportions and differences between the two groups were evaluated using the Chi-squared (χ2) statistic test or Fischer exact test, when applicable. Deviations from Hardy–Weinberg equilibrium (HWE) were assessed by the goodness-of-fit χ2 test. The distribution of alleles and genotypes of *COL1A1* and *COL1A2* polymorphisms were derived by gene counting and the difference of the frequencies between the groups were evaluated using the χ2 test or the Fisher’s exact test, when appropriate. The haplotype patterns and linkage disequilibrium coefficients (D′: imbalance degree in module and R^2^: correlation degree) were inferred using Haploview (https://haploview.software.informer.com/4.2/), as previously described [37]. The association analysis between epidemiological, sports, and training characteristics as much as of the polymorphisms with ACL rupture was estimated by the odds ratio (OR) with a 95% confidence interval (95% CI). Multivariable logistic regression model by the stepwise method was performed to evaluate the possible associations and to set the final analysis model each variable was introduced considering its biological plausibility and univariate statistical significance [significance level – input: *P* ≤ 0.20 and output: *P* ≤ 0.05]. The statistically significant difference was set at *P* ≤ 0.05. All analyses were performed using the Statistical Package for Social Sciences (SPSS Inc., Chicago, IL, USA, version 20.0). The sample size (*N* = 338) was appropriated to detect differences between case and control groups, assuming an odds ratio of 2.0 with a power of 0.8 and 5% type I error (Epi Info 7, version 7.1.3., https://www.cdc.gov/epiinfo/por/pt_pc.html).

## Results

We observed a total of 146 ACL rupture cases, being 67 (45.9%) due to a contact injury, 67 (45.9%) were non-contact, and 12 (8.2%) did not inform about the nature of the injury. In addition, 102 (70.8%) underwent an ACL reconstruction surgery, while 42 (29.2%) were waiting for the surgical procedure to be scheduled. Additional relevant ACL rupture-related information and its comparison between contact and non-contact injury subgroups are displayed in Table 2, and non-contact injuries were more likely (~ 2.5 times) to occur during training than contact injuries (Table 2).

**Table 2:** Clinical characteristics of the ACL injury-related information in the athletes

Variables	Injured Group (***N*** = 146)^a^	Contact ACL injury (***N*** = 67)	Non-contact ACL injury (***N*** = 67)	***P*** – value^b^	OR (CI 95%)
	N (%)				
**ACL injury sporting event**^c^					
Competition	72 (54.5)	42 (64.6)	30 (45.5)	0.06	1^d^
Training	47 (35.7)	17 (26.2)	30 (45.5)		2.47 (1.16–5.27)
Competition and Training	13 (9.8)	6 (9.2)	6 (9.0)		1.40 (0.41–4.76)
**Post-training knee pain**					
No	69 (47.3)	29 (43.3)	32 (47.8)	0.60	1^d^
Yes	77 (52.7)	38 (56.7)	35 (52.2)		0.83 (0.42–1.65)
**Surgery number**^e^					
1	73 (78.5)	37 (82.2)	36 (75.0)	0.40	1^d^
≥ 2	20 (21.5)	8 (17.8)	12 (25.0)		1.54 (0.56–4.21)
**ACL injury recurrence**^f^					
No	113 (83.7)	55 (82.1)	58 (86.6)	0.48	1^d^
Yes	22 (16.3)	12 (17.9)	9 (13.4)		0.71 (0.28–1.82)
**Time away from training**					
1 to 3 months	43 (29.5)	22 (32.8)	19 (28.4)	0.87	1^d^
4 to 6 months	37 (25.3)	17 (25.4)	17 (25.4)		1.16 (0.47–2.88)
7 to 9 months	38 (26.0)	15 (22.4)	19 (28.4)		1.47 (0.59–3.66)
≥ 10 months	28 (19.2)	13 (19.4)	12 (17.9)		1.07 (0.39–2.89)

OR is the Odds ratio; CI is the confidence interval

^a^Contact or non-contact ACL injury-related information was obtained from 134 athletes

^b^*P*-value ≤0.05 was obtained through the Chi-squared Test (Pearson *P*-value) to compare contact and non-contact ACL injuries

^c^Information was obtained from 132 athletes (65 contact ACL injuries, 66 non-contact ACL injury and 1 without information about contact or non-contact ACL injury)

^d^Reference value

^e^Information was obtained from 93 athletes (45 contact ACL injury and 48 non-contact ACL)

^f^Information was obtained from 135 athletes

The ACL rupture cases (both contact and non-contact subgroups) were significantly older than controls (27.2 ± 6.1 vs. 23.0 ± 5.0 years old, *P =* 0.002), with higher sports practice time (12.4 ± 6.7 vs. 8.3 ± 5.8 years, *P =* 0.07), and weekly training hours (15.5 ± 9.8 vs. 12.3 ± 6.7 h, *P =* 0.01) (Table 3). Table 3 describes the demographic, sport, and training characteristics of the studied athletes. In summary, all variables were analyzed to identify possible confounding variables of the true association between *COL1A1* and *COL1A2* SNPs and the ACL rupture. Initially, for the analysis of injured group, the variables age (*P* < 0.001), sex (*P* = 0.11), sport modality (*P* = 0.01), training location (*P* = 0.05), years of training (*P* = 0.03) were inserted in the logistic regression model. After the stepwise regression method, the variables age, sport modality, training location, and years of training remained in this model. On the other hand, for analysis of non-contact ACL rupture, only age, sport modality, and training location were considered confounding variables. The variables age, sport modality, training location, and years of training were associated with ACL rupture when considering injured group for the analysis. Likewise, the age, sport modality, and training location remained associated with only non-contact ACL rupture in athletes (Table 3).

**Table 3:** Epidemiological, sport, and training characteristics of the athletes

Variables	Control (***N*** = 192)	Injured Group (***N*** = 146)	***P*** – value^a^	Adjusted OR (CI 95%)^b^	Non-contact ACL (***N*** = 67)	***P*** – value^a^	Adjusted OR (CI 95%)^c^
	N (%)				N (%)		
**Age (years)**^d^							
18 to 20	85 (44.3)	21 (14.4)	< 0.001	1^e^	10 (14.9)	< 0.001	1^e^
21 to 24	47 (24.5)	33 (22.6)		3.64 (1.80–7.38)	10 (14.9)		2.38 (0.88–6.45)
25 to 29	35 (18.2)	44 (30.1)		5.31 (2.61–10.80)	19 (28.4)		5.69 (2.29–14.12)
30 to 45	25 (13.0)	48 (32.9)		7.37 (3.45–15.75)	28 (41.8)		12.52 (5.00–31.38)
**Sex**							
Female	63 (32.8)	53 (36.3)	0.11	1^e^	20 (29.9)	0.87	1^e^
Male	129 (67.2)	93 (63.7)		0.64 (0.38–1.10)	47 (70.1)		0.94 (0.47–1.88)
**BMI (Kg/m**^**2**^**)**							
< 25.0	123 (64.0)	77 (52.7)	0.28	1^e^	37 (55.2)	0.99	1^e^
25.0 a 29.9	56 (29.2)	54 (37.0)		1.20 (0.70–2.08)	23 (34.3)		1.00 (0.50–2.03)
≥ 30	13 (6.8)	15 (10.3)		2.07 (0.83–5.17)	7 (10.5)		1.28 (0.37–4.42)
**Skin colour**^f^							
White	72 (37.9)	52 (37.1)	0.76	1^e^	26 (40.0)	0.52	1^e^
Intermediate	62 (32.6)	53 (37.9)		1.12 (0.62–2.01)	21 (32.3)		0.86 (0.39–1.86)
Black	51 (26.9)	31 (22.1)		0.83 (0.42–1.61)	15 (23.1)		0.95 (0.41–2.24)
Others	5 (2.6)	4 (2.9)		1.57 (0.35–7.01)	3 (4.6)		3.31 (0.63–17.39)
**Sport modality**							
Rugby	79 (41.1)	38 (26.0)	0.01	1^e^	15 (22.4)	0.03	1^e^
Soccer	43 (22.4)	38 (26.0)		1.56 (0.73–3.32)	22 (32.8)		3.12 (1.33–7.36)
Combat	20 (10.4)	29 (19.9)		4.07 (1.46–11.38)	11 (16.4)		5.57 (1.45–21.48)
Handball	13 (6.8)	15 (10.3)		5.92 (1.64–21.30)	4 (6.0)		4.40 (0.85–22.86)
Water polo	21 (11.0)	6 (4.1)		2.62 (0.61–11.27)	4 (6.0)		7.16 (1.14–45.09)
Others	16 (8.3)	20 (13.7)		6.57 (1.98–21.31)	11 (16.4)		10.22 (2.08–50.24)
**Training location**							
Grass	121 (63.0)	84 (57.5)	0.005	1^e^	41 (61.2)	0.01	1^e^
Sports court	13 (6.8)	15 (10.3)		0.46 (0.11–1.93)	6 (8.9)		0.07 (0.01–0.78)
Mat	20 (10.4)	29 (19.9)		0.60 (0.25–1.42)	11 (16.4)		0.28 (0.08–1.00)
Pool	24 (12.6)	7 (4.8)		0.09 (0.02–0.42)	5 (7.5)		0.04 (0.01–0.42)
Beach	7 (3.6)	7 (4.8)		0.30 (0.07–1.34)	2 (3.0)		0.05 (0.01–0.60)
Others	7 (3.6)	4 (2.7)		0.04 (0.01–0.36)	2 (3.0)		0.01 (0.01–0.14)
**Years of training** ^d^							
≤ 5	69 (35.9)	24 (16.4)	0.03	1^e^	11 (16.5)	0.17	1^e^
6 to 10	62 (32.3)	39 (26.7)		1.81 (0.93–3.54)	21 (31.3)		2.25 (0.91–5.57)
11 to 15	41 (21.4)	42 (28.8)		2.51 (1.24–5.06)	14 (20.9)		1.72 (0.64–4.62)
> 15	20 (10.4)	41 (28.1)		2.65 (1.20–3.54)	21 (31.3)		2.79 (1.02–7.64)
**Weekly training hours** ^d^							
≤ 8	63 (32.8)	32 (21.9)	0.40	1^e^	15 (22.4)	0.29	1^e^
9 to 12	58 (30.2)	37 (25.3)		0.94 (0.47–1.91)	16 (23.9)		1.08 (0.43–2.67)
13 to 18	38 (19.8)	36 (24.7)		1.45 (0.71–2.96)	15 (22.4)		1.51 (0.60–3.83)
> 18	33 (17.2)	41 (28.1)		1.57 (0.74–3.36)	21 (31.3)		2.26 (0.89–5.73)

OR is the Odds ratio; CI is the confidence interval

^a^*P*-value ≤0.05 was obtained through the Chi-squared Test (Pearson *P*-value)

^b^OR adjusted by age, sport modality, training location and years of training

^c^OR adjusted by age, sport modality, and training location

^d^Values are categorized according to the quartile distribution of the total study population

^e^Reference value

^f^Information was obtained from 230 athletes (control = 190, injured group = 140, non-contact ACL = 65)

The *COL1A1* (*rs*1107946) and *COL1A2* (rs412777, rs42524, rs2621215) SNPs were in Hardy–Weinberg equilibrium in the overall studied athletes and each group (case and control). Association analyses of the *COL1A1* and *COL1A2* SNPs with ACL rupture are shown in Table 4. After adjustment for confounding factors (age, sport modality, and training location) variant genotype *CC* (rs42524), allele and genotypes variants *G* and *GG* (rs2621215) of the *COL1A2* gene were associated with increased risk of non-contact ACL rupture. However, no significant differences were detected in allele or genotype distribution of all SNPs (*COL1A1* rs1107946 and *COL1A2* rs412777, rs42524, rs2621215) comparing control and injured group of the ACL tear (adjustment for age, sport modality, training location, and years of training).

**Table 4:** Association analyses of the *COL1A1* and *COL1A2* polymorphisms with ACL rupture

SNPs	Control (*N* = 192)	Injured Group (*N* = 146)	*P* – value^a^	Adjusted OR (CI 95%)^b^	Non-contact ACL (*N* = 67)	*P* – value^a^	Adjusted OR (CI 95%)^c^
	***N*** (%)				N (%)		
*COL1A1*							
rs1107946^e^							
*GG*	110 (57.9)	92 (63.4)	0.71	1^d^	45 (67.2)	0.69	1^d^
*GT*	65 (34.2)	43 (29.7)		0.82 (0.47–1.40)	17 (25.3)		0.77 (0.37–1.57)
*TT*	15 (7.9)	10 (6.9)		1.10 (0.43–2.81)	5 (7.5)		1.22 (0.37–3.94)
*G*	285 (75.0)	227 (78.3)	0.96	1^d^	107 (79.9)	0.93	1^d^
*T*	95 (25.0)	63 (21.7)		0.99 (0.65–1.50)	27 (20.1)		1.02 (0.59–1.78)
*COL1A2*							
rs412777^f^							
*AA*	77 (41.0)	67 (46.2)	0.33	1^d^	30 (44.8)	0.40	1^d^
*AC*	87 (46.3)	62 (42.8)		0.81 (0.48–1.36)	31 (46.3)		1.03 (0.53–1.99)
*CC*	24 (12.8)	16 (11.0)		0.55 (0.25–1.24)	6 (8.9)		0.49 (0.17–1.47)
*A*	241 (64.1)	196 (67.6)	0.16	1^d^	91 (67.9)	0.34	1^d^
*C*	135 (35.9)	94 (32.4)		0.77 (0.53–1.11)	43 (32.1)		0.80 (0.50–1.28)
rs42524^g^							
*GG*	128 (67.0)	88 (60.7)	0.26	1^d^	41 (61.2)	0.09	1^d^
*GC*	59 (30.9)	48 (33.1)		1.22 (0.73–2.04)	21 (31.3)		1.19 (0.60–2.35)
*CC*	4 (2.1)	9 (6.2)		2.85 (0.76–10.75)	5 (7.5)		5.73 (1.22–26.95)
*G*	315 (82.5)	224 (77.2)	0.17	1^d^	103 (76.9)	0.10	1^d^
*C*	67 (17.5)	66 (22.8)		1.35 (0.88–2.06)	31 (23.1)		1.57 (0.92–2.69)
rs2621215^f^							
*TT*	116 (61.7)	78 (53.8)	0.18	1^d^	35 (52.2)	0.06	1^d^
*TG*	64 (34.0)	56 (38.6)		1.38 (0.83–2.30)	25 (37.3)		1.32 (0.67–2.59)
*GG*	8 (4.3)	11 (7.6)		2.36 (0.83–6.68)	7 (10.4)		4.29 (1.26–14.61)
*T*	296 (78.7)	212 (73.1)	0.08	1^d^	95 (70.9)	0.04	1^d^
*G*	80 (21.3)	78 (26.9)		1.42 (0.96–2.13)	39 (29.1)		1.69 (1.02–2.81)

OR is the Odds ratio; CI is the confidence interval

^a^*P*-value ≤0.05 was obtained through the Chi-squared Test (Pearson *P*-value)

^b^OR adjusted by age, sport modality, training location and years of training

^c^OR adjusted by age, sport modality, and training location

^d^Reference value

^e^Successful genotyping was 335 samples

^f^Successful genotyping was 333 samples

^g^Successful genotyping was 336 samples

**Fig. 1:**
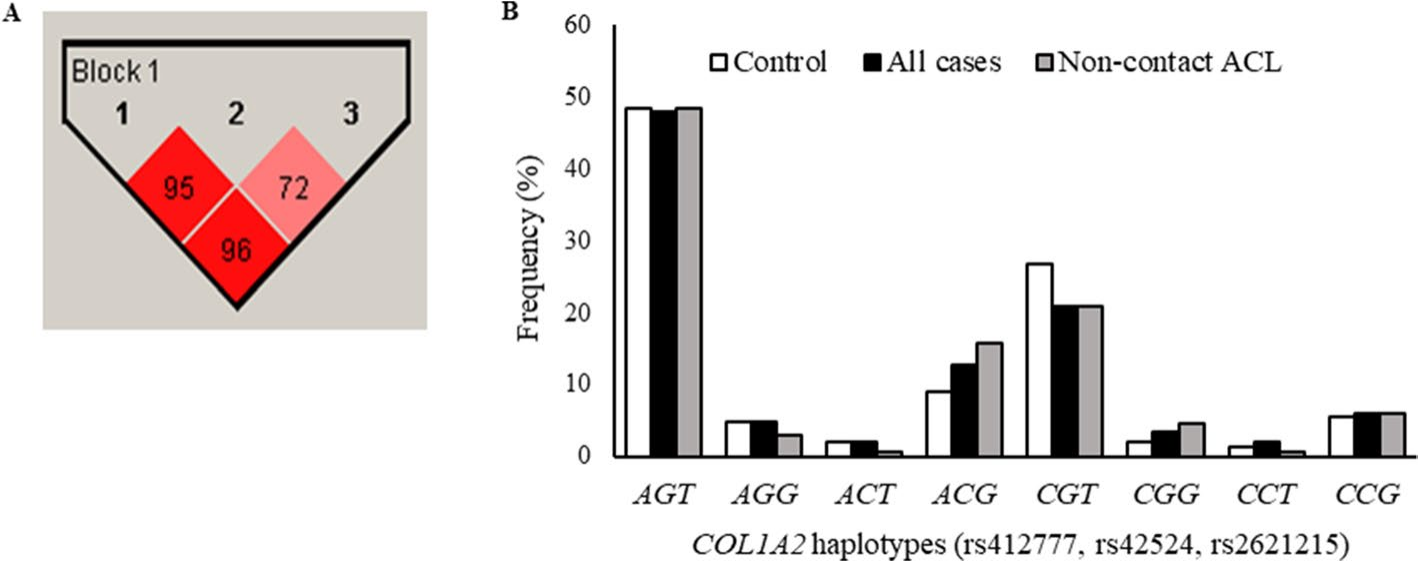
Haplotype analysis for *COL1A2* (rs412777, rs42524, rs2621215) SNPs in Brazilian athletes. **A**. Number in boxes indicates decimal places of D′. **B**. Haplotype distributions between injured group and non-contact ACL injury compared to control group

Haplotypes of the *COL1A2* gene were determined for all athletes. The results revealed that SNPs rs412777, rs42524, rs2621215 were in strong linkage disequilibrium, forming a single haploblock (Fig. 1A). Eight haplotypes could be inferred and *AGT* haplotype was considered wildtype/reference haplotype due to present the highest frequency in the study population (*N* = 320, 47.3%). However, after multivariate analysis, no significant differences were detected in haplotype frequencies between controls and injured group of ACL rupture, neither between controls, nor non-contact injury (Fig. 1B).

On the other hand, a combined analysis of the four studied *COL1A1* and *COL1A2* SNPs was performed to investigate whether the presence of more than one SNP would increase the risk of developing the ACL rupture (Table 5). After adjustment for age, sport modality, training location, and years of training, combined variant genotype *COL1A1 GT* or *TT* (rs1107946) and the wild-type genotypes of the three *COL1A2* (rs412777 *- AA,* rs42524 *- GG,* rs2621215 *- TT*) SNPs were negatively associated with ACL rupture compared with the reference wild-type genotypes *COL1A1* (rs1107946 - *GG*) and *COL1A2* (rs412777 *- AA,* rs42524 *- GG,* rs2621215 *- TT*).

**Table 5:** Combined analysis of the *COL1A1* and *COL1A2* polymorphisms and the risk of developing ACL injury

*COL1A1* and *COL1A2*	Control (*N* = 192)	Injured Group (*N* = 146)	*P* – value^a^	Adjusted OR (CI 95%)^b^	Non-contact ACL (*N* = 67)	*P* – value^a^	Adjusted OR (CI 95%)^c^
rs1107946 + (rs412777 / rs4252 / rs2621215)^e^	**N (%)**				**N (%)**		
*WT /* 3 *WT*	13 (7.0)	16 (11.0)	0.32	1^d^	8 (11.9)	0.34	1^d^
*WT / 1 VAR*	58 (31.2)	37 (25.5)		0.39 (0.15–1.02)	16 (23.9)		0.43 (0.13–1.42)
*WT / 2 VAR*	24 (12.9)	26 (17.9)		0.58 (0.21–1.65)	16 (23.9)		0.91 (0.26–3.17)
*WT / 3 VAR*	12 (6.4)	13 (9.0)		0.65 (0.20–2.15)	5 (7.5)		0.62 (0.13–2.94)
*VAR / 3 WT*	13 (7.0)	8 (5.5)		0.25 (0.07–0.96)	4 (6.0)		0.33 (0.06–1.74)
*VAR / 1 VAR*	38 (20.4)	20 (13.8)		0.41 (0.14–1.15)	7 (10.4)		0.35 (0.09–1.40)
*VAR / 2 VAR*	20 (10.8)	21 (14.5)		0.79 (0.26–2.35)	8 (11.9)		0.96 (0.24–3.90)
*VAR / 3 VAR*	8 (4.3)	4 (2.8)		0.45 (0.10–2.13)	3 (4.5)		1.46 (0.25–8.46)

OR is the Odds ratio; CI is the confidence interval

^a^*P*-value ≤0.05 was obtained through the Chi-squared Test (Pearson *P*-value)

^b^OR adjusted by age, sport modality, training location and years of training

^c^OR adjusted by age, sport modality, and training location

^d^Reference value

^e^ Successful combined genotypes were 331 samples

## Discussion

The two main findings of this study were that *COL1A2* SNPs (rs42524 and rs2621215) increased the non-contact ACL tear risk and the rs1107946 *COL1A1* SNP was associated with ACL tear only when the three *COL1A2* SNPs (rs412777, rs4252, and rs2621215) were wildtype. These results are in line with the previous studies suggesting that genetic contributions should be considered as an intrinsic and non-modifiable risk factor for ACL rupture susceptibility [9], and that polymorphisms in collagen genes may contribute to the development of this injury in both sexes [25, 27, 28].

It is currently accepted that ACL tears have multifactorial etiology, represent ~ 50–60% of knee injuries [38] and usually (70–84%) occur through low-energy non-contact events [39, 40]. In the present study, age, sport modality, training location, and time of practice were associated with an ACL tear in general. However, in the non-contact ACL tear subgroup, only age, sport modality, and training location remained associated with the injury. Those were already well-described risk factors associated with ACL rupture [40–44]. Moreover, our results reinforce the higher risks to ACL rupture according to sport modality and training location – for example, soccer (3-fold) for soccer players and grass [45].

We observed that the *COL1A2* rs2621215 *GG* genotype was associated with non-contact ACL rupture risk (4-fold). This SNP is in intron 46 and can interact with other functional polymorphisms and affect the removal of these gene introns [46]. Thus, the presence of the variant allele *G* rs2621215 could produce more rigid collagen and increase the risk of ACL rupture susceptibility. Also, *COL1A2* rs42524 *G > C*, located in exon 28, increased the risk of non-contact ACL (~ 6-fold). This coding SNP causes alanine to proline substitution at amino acid residue 459 (Ala459Pro) in the Y-position of the Gly-X-Y repeat of the collagen I α2 helical region [46, 47], and the change in protein conformation affects collagen type I stiffness, which the Pro-459 variant amino acid is more rigid than that the Ala-459 [46]. Theoretically, more rigid collagen might produce stiffer (or less compliant) ACL, consequently reducing the tissue capacity to dissipate mechanical energy and increasing the chance of failure with reduced external forces [48].

Interestingly, the combined variant genotype *COL1A1* rs1107946 with wildtype genotypes of the three *COL1A2* (rs412777, rs42524, rs2621215) SNPs produced a protective factor to the ACL tear. The rs1107946 located on the promoter region of the *COL1A1* gene (*−1997G > T*) and the presence of variant allele *G* has a higher transcriptional activity than the wild-type allele *T* [25, 34]. Also, this finding agrees with Ficek et al., who did not observe any association of the *COL1A1* rs1107946 with ACL rupture, except when it was combined with the *COL1A1* rs1800012 SNP [25]. Sivertsen et al. analyzed six collagen genes polymorphisms (*COL1A1, COL3A1, COL5A1*, and *COL12A1*), including *COL1A1* rs1107946, and did not observe an association between ACL rupture risk and selected polymorphisms in female elite athletes from Norway and Finland. However, no combined genotype analysis was conducted [49]. Here, the cumulative effect of the *COL1A1* and *COL1A2* SNPs suggested a gene-gene interaction that might change the collagen type 1 α1/α2 chains ratio, thus predisposing the ACL rupture.

Hence, genetic investigations may help in the understanding of non-contact ACL ruptures [40]. This was the first study investigating a genetic combination of the *COL1A1* and *COL1A2* polymorphisms to the susceptibility to a non-contact ACL tear in athletes. Besides the several strong points, like adequate sample size and all injured subjects were diagnosed confirmed by a gold standard medical image exam or underwent an ACL surgical reconstruction, some limitations should be kept in mind. There is always the risk of recall bias in self-reported questionaries, twelve subjects did not remember the injury nature (contact or non-contact) and were excluded from the subgroup analysis (Table 2), and genotyping failure of some samples. However, we believe those limitations did not affect the statistical outcomes.

Investigating the link between ACL rupture and genetic variants is an important subject to a better elucidation of injury etiology and risk factors. The present results can be used to build a database from different populations to identify the impacts of genetics SNPs on ACL rupture risk [50]. Therefore, the knowledge regarding possible individual predisposing factors is essential to understand the molecular mechanism of ACL rupture and improve the disease prognosis by screening and prevention protocols. Due to the loss of physical performance, sports ability of their subjects, and high treatment costs, the genetic information can assist in ACL tear susceptibility identification, as contributing to individualized training and surveillance for monitoring the at-risk individuals. However, caution should be considered when generalization those results to other populations, and further studies should be carried on this topic.

## Conclusion

In summary, *COL1A2* rs42524 and rs2621215 SNPs were associated with non-contact ACL risk, which represents an advance in understanding the etiology of disease. The combined analysis of *COL1A1*-*COL1A2* genotypes suggests a gene-gene interaction in ACL rupture susceptibility. These findings suggest that polymorphism screening could identify athletes with a higher risk of non-contact ACL rupture, which could benefit from specific training and surveillance protocols to prevent lesions.

## Data Availability

The datasets used and/or analyzed during the current study are available from the corresponding author on reasonable request.

## Abbreviations

ACL: Anterior cruciate ligament
95% CI: 95% Confidence interval
χ2: Chi-square
BMI: Body mass index
*COL1A1*: Collagen type I alpha 1 chain
*COL1A1*: Collagen type I alpha 2 chain
HWE: Hardy–Weinberg equilibrium
OR: Odds ratio
PCR: Polymerase chain reaction
R^2^: Degree of correlation
SD: Standard deviation
SPSS: Statistical Package for Social Sciences
